# The value of shear wave elasticity and shear wave dispersion imaging to evaluate the viscoelasticity of renal parenchyma in children with glomerular diseases

**DOI:** 10.1186/s12882-023-03357-1

**Published:** 2023-10-19

**Authors:** Shixiang Yao, Yingying Cai, Shanshan Hu, Xiao Liu, Xia Gao, Guanyu Li, Hongying Wang, Hongkui Yu

**Affiliations:** 1 Department of Ultrasonography, Guangzhou Women and Children’s Medical Center, Guangzhou Medical University, No.9, Jinsui Road, Guangzhou, Guangdong China; 2 The Sixth Clinical Medical College of Guangzhou University of Chinese Medicine, Shenzhen, Guangdong China; 3 Department of Ultrasonography, Shenzhen Hospital of Guangzhou University of Chinese Medicine, Shenzhen, Guangdong China; 4 Nephrology department, Guangdong Provincial Clinical Research Center for Child Health, Guangzhou Women and Children’s medical center, Guangzhou city, China

**Keywords:** Child, Kidney, Elasticity imaging techniques, Shear wave dispersion imaging, Viscoelasticity

## Abstract

**Background:**

To study the value of shear wave elasticity and shear wave dispersion imaging to evaluate the viscoelasticity of renal parenchyma in children with glomerular diseases.

**Methods:**

Forty-three children with glomerular diseases were prospectively evaluated by shear wave elasticity (SWE) and shear wave dispersion imaging (SWD); 43 healthy volunteers served as the control group. The shear wave velocities (SWV) and the dispersion slopes were measured at the upper, middle, and lower poles of both kidneys. The analysis of mean SWV and mean dispersion slope in control and patient groups was used to further evaluate the value of SWE and SWD in the viscoelasticity of renal parenchyma in children with glomerular disease.

**Results:**

The mean SWV in children with glomerular disease was higher than that in the healthy control group (1.61 ± 0.09 m/s vs. 1.43 ± 0.07 m/s, p < 0.001). Compared with healthy group, the mean dispersion slope in children with glomerular disease was significantly increased (13.5 ± 1.39 (m/s)/kHz vs. 12.4 ± 1.40 (m/s)/kHz, p < 0.001). Correlation analysis showed absence of correlation between the SWV and dispersion slope of occult blood, serum creatinine, 24-h urine protein, blood albumin, BMI and ROI box depth of children with glomerular disease.

**Conclusions:**

The present study shows that it is feasible to use SWE and SWD to evaluate the difference of viscoelasticity of the renal parenchyma between healthy children and those with glomerular disease.

## Introduction

Glomerular disease is a common cause of kidney disease in children and accounts for 5–14% of chronic kidney disease (CKD) among children worldwide and is also the main cause of end-stage kidney disease in China [1–3]. Glomerular diseases refer to a group of symptoms with similar clinical manifestations, such as hematuria, proteinuria, and hypertension, but different etiology, pathogenesis, pathological changes, course, and prognosis. The lesions typically affect the glomeruli of both kidneys. Nephrotic syndrome is one of the most common manifestations of glomerular disease in children, affecting 2–7 children per 100,000 population [4].

Renal biopsy is the gold standard for diagnosing glomerular disease, which can determine the pathological type, severity and activity of the lesion, but also with contraindications and possible complications such as bleeding and infection [5]. Traditional ultrasound diagnosis of renal diseases requires related factors such as changes of parenchymal echo and size. These changes indicate likely renal dysfunction. Therefore, it is necessary to identify a non-invasive detection method that can provide reference data for clinical practice at an early stage.

The measurement of soft tissue elasticity is of great significance in the diagnosis of different diseases such as those affecting the liver, breast, thyroid, prostate, and other related conditions [6–9]. The quantitative measurements of tissue stiffness can be obtained by using shear wave elastography (SWE). When combined with conventional ultrasound imaging, the method uses acoustic radiation forces to generate transversely propagating shear waves that can be tracked to determine their velocity. However, in addition to elasticity, there is also viscosity, which plays an important role in the propagation of shear waves. However, in most ultrasound elastography methods, only tissue elasticity is quantified, while tissue viscosity is ignored [10]. It is well known that dispersion is related to the frequency dependence of shear waves propagation velocity and the attenuation of shear waves propagation in the viscous component. If the tissue is diffuse, the velocity of the shear waves and the attenuation of the shear waves will increase with frequency [11]. Shear waves dispersion (SWD) can be used to estimate the dispersion slope of the shear waves’ speed versus frequency to evaluate changes in tissue viscosity [12]. Therefore, in this study, we aimed to quantitatively measure elasticity-related shear waves velocity (SWV) and viscosity-related dispersion slope in the kidney.

The purpose of this study is to explore the feasibility of evaluating the difference of viscoelasticity of the renal parenchyma between healthy children and children those with glomerular disease by SWE and SWD.

## Patients and methods

### Study subjects

**Fig. 1:**
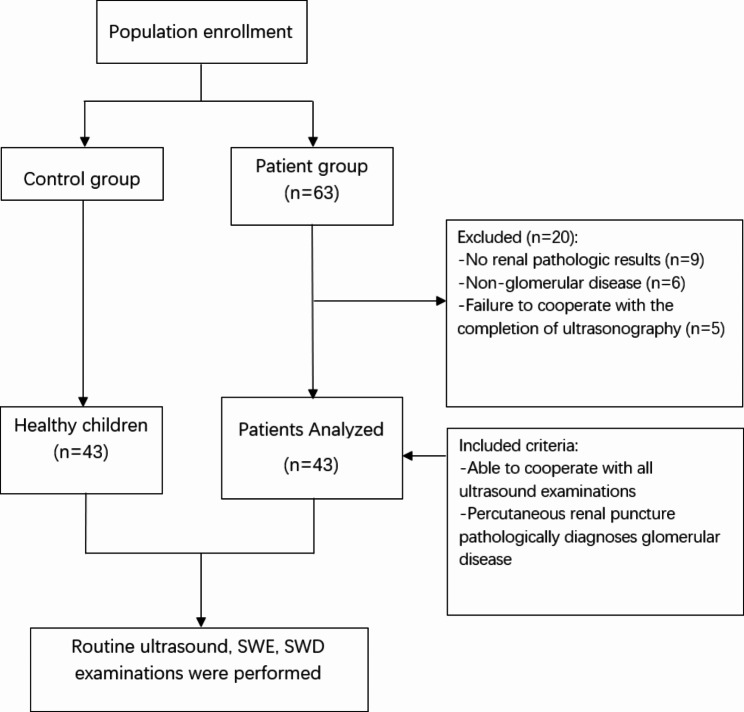
Study design and participants

This study was approved by the Ethics Committee of Guangzhou Women and Children’s Medical Center(2021169A01). Parents of all the included subjects signed informed consent. The patient group includes children who received treatment at our hospital between July 2021 and April 2022. The inclusion criteria for the patient group were as follows: first, children who have undergone conventional renal ultrasound, SWE, and SWD examination; second, children who have undergone percutaneous renal biopsy and obtained a confirmed pathological diagnosis of glomerular disease (Fig. 1).

Patients were excluded if they fulfilled one of the following criteria: (i) unable to cooperate or sustain all ultrasound examinations; (ii) incomplete renal biopsy; and (iii) pathological results of renal biopsy not conclusive of glomerular disease.

The control group comprised children who were treated in our hospital from July 2021 to April 2022, but did not have any kidney disease or related diseases affecting renal function.

### Technique

**Fig. 2:**
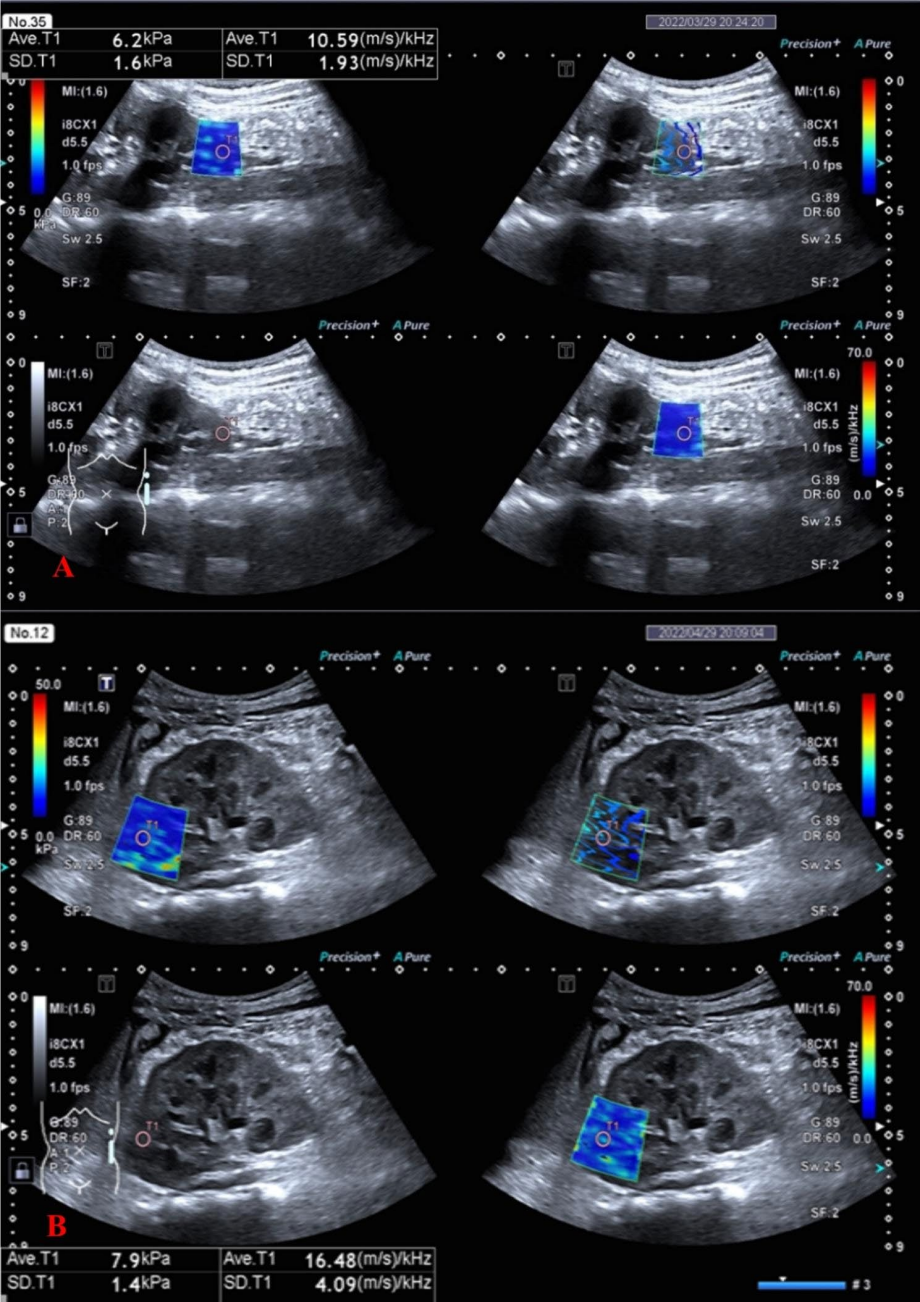
**A**, Measurement of kidney shear wave speed with ARFI elastography on healthy kidney. **B**, Measurement of kidney shear wave speed with ARFI elastography on glomerular diseases. QuadView four mode can simultaneously display short-wave velocity or short-wave elasticity (velocity map, elasticity map), short-wave arrival time contour (propagation map), gray level and dispersion slope (dispersion map)

SWE measurements were performed by two radiologists using an Aplio i900 (Canon Medical Systems Corporation) ultrasound system software, with a 1- to 8-MHz i8CX1 convex array probe. All patients were scanned in the supine position after urination. Routine ultrasonography was first performed to measure and record the long diameter of both kidneys. Then, the conventional ultrasound mode was switched manually to the SWE mode and the SWD could be activated automatically in SWE mode. The region of interest (ROI: 5 × 5 mm) was placed vertically in the renal parenchyma of the upper pole, middle pole, and lower pole. The ROI should avoid structures such as vascular structures. SWE and SWD measurements were performed during breath hold if the child could control breathing. The Quad-View four mode can simultaneously display short-wave velocity (velocity map); short-wave arrival time contour (propagation map); gray level; and dispersion slope (dispersion map) (Fig. 2). The isochronous arrival curve displayed in the propagation map is a quality control indicator to show whether the shear wave data is stable. If the isochronous curve is regular and the interval is constant, it is considered credible. If the curve is disorderly, i.e., the shear wave propagation is disordered, the measurement is considered unreliable and the measurement fails. In the SWE measurement image, “red indicates higher elasticity, and blue indicates lower elasticity”; in the SWD measurement images, “red indicates higher viscosity and blue indicates lower viscosity”. The shear wave velocity is displayed in m/s, and the dispersion slope is displayed in (m/s)/kHz. The image acquisition and measurement data were repeated 5 times, and the mean value was recorded and calculated.

To assess inter-observer reliability, kidney measurements were performed by two different examiners on 18 randomly selected subjects. Both examiners had more than10 years’ experience in ultrasound and were trained in SWE and SWD technology. The SWV and dispersion slope of 18 patients were measured by Bland–Altman curve. For the remaining patients, SWE and SWD measurements were taken by examiner 1, and the final mean SWV and mean dispersion slope were calculated based on the first examiner’s measurements.

### Statistical analysis

Statistical analysis was performed using SPSS (version 25.0, IBM Corporation, Armonk, NY, USA). Data are presented as Mean ± SD and as median and range (from minimum to maximum). The distribution of quantitative data was determined using the Shapiro–Wilk test for normality. To compare two independent groups, the *t*-test and Mann–Whitney U test were used for normally and non-normally distributed data, respectively. Pearson’s correlation was used for normally distributed data, and Spearman correlation was used for correlation analysis for non-normally distributed data. Correlation analysis was performed on the relationship between the mean SWV and mean dispersion slope of both kidneys and serum creatinine, 24-h urine protein, BMI, and ROI box depth. To assess inter-observer variability, the Bland–Altman method was used. The reliability of the measurement results was evaluated by the correlation coefficient between groups. P < 0.05 was considered to indicate statistically significant differences. The maximum cut-off value was considered as the diagnostic cut-off value by using ROC curves and combining sensitivity with specificity, and the SWV and dispersion slope cut-off values to distinguish the disease group from the control group was estimated.

## Results

**Fig. 3:**
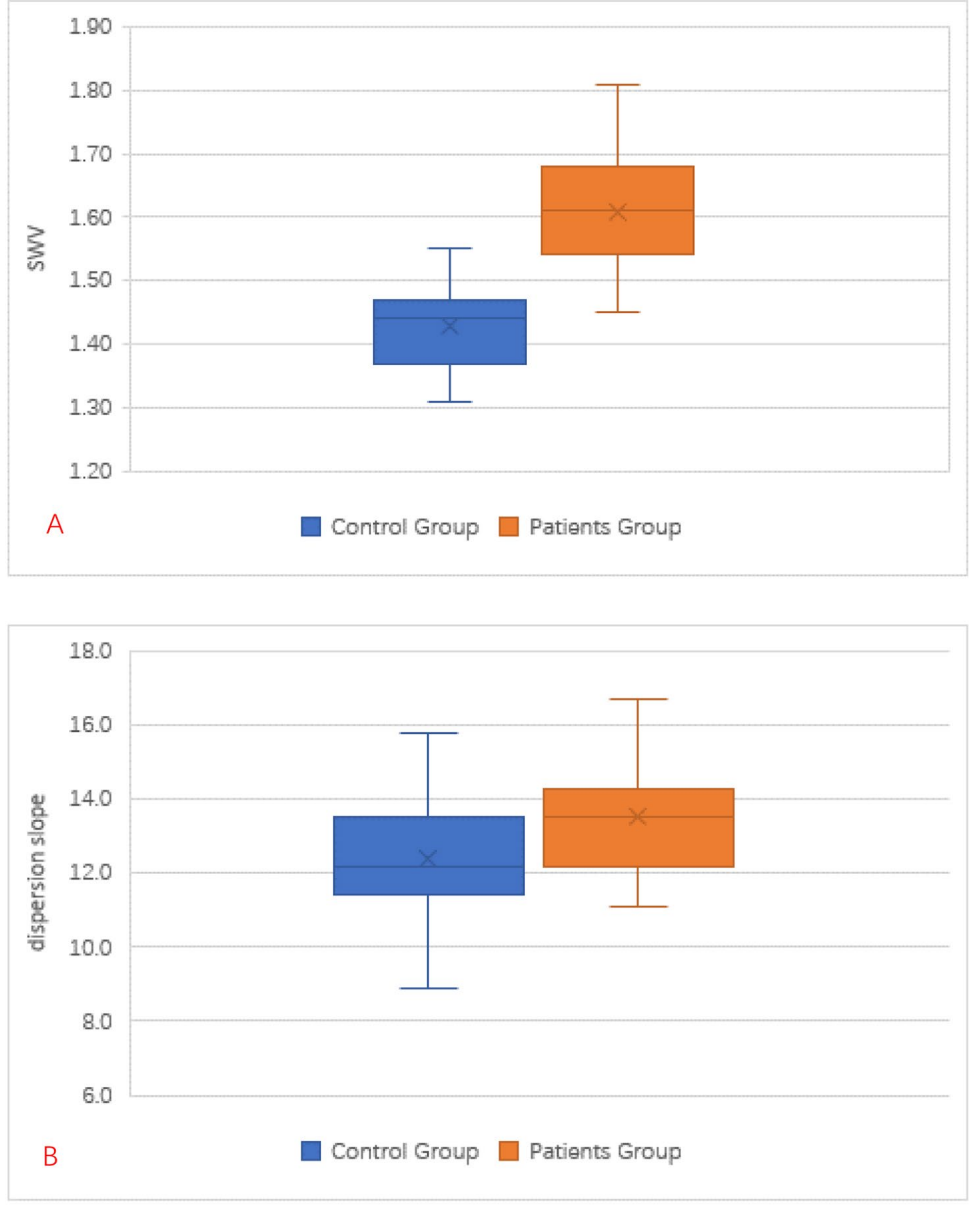
**A** shows the difference in renal shear wave velocity values assessed by SWE technique between children with glomerular disease and the control group; **B** shows the difference in renal frequency slope assessed by SWD technique between children with glomerular disease and the control group

Forty-three patients comprised the patient group (27 boys and 16 girls, mean age: 8.3 ± 3.63 [2.0–13.7] years). Forty-three children (22 boys and 21 girls, mean age: 8.2 ± 3.62 [2.1–13.6] years) were selected as the control group. All patients in the study were compared with controls, and no significant difference was found between the two groups with respect to demographic parameters such as age, sex, height, weight, and BMI (p > 0.05) (Table 1). The mean SWV of the kidneys of 43 children with glomerular disease was 1.61 ± 0.09 m/s, while the mean SWV of healthy children was 1.43 ± 0.07 m/s (p < 0.001). The mean dispersion slope of children with glomerular disease was 13.5 ± 1.39 (m/s)/kHz, and the healthy control group was 12.4 ± 1.40 (m/s)/kHz (p < 0.001) (Table 2; Fig. 3).

**Table 1:** Demographic Data and Characteristics of Study Subjects

Characteristic	Patients with glomerular Diseases (n = 43)	Control Subjects (n = 43)	P
Age(y)			0.99
Mean ± SD	8.3 ± 3.63	8.2 ± 3.62	
Median(range)	8.3 (2.0-13.7)	8.4(2.1–13.6)	
Gender			0.28
Female	16	21	
Male	27	22	
Height(m)			0.75
Mean ± SD	1.26 ± 0.23	1.29 ± 0.23	
Median(range)	1.23(0.83–1.55)	1.33(0.85–1.72)	
Weight(kg)			0.80
Mean ± SD	28.4 ± 11.60	27.7 ± 11.79	
Median(range)	27.9(12.0–55.0)	28.0(10.0–57.0)	
Body mass index ^a^			0.12
Mean ± SD	17.16 ± 2.98	15.89 ± 2.71	
Median(range)	16.62(13.07–26.06)	15.75(8.26–25.92)	

^a^: Weight in kilograms divided by the square of height in meters

**Table 2:** Ultrasound findings of Study Subjects

Characteristic	Patients with glomerular Diseases (n = 43)	Control Subjects (n = 43)	P
Depth (cm)			P = 0.80
Mean ± SD	4.4 ± 0.64	4.4 ± 0.54	
Median(range)	4.3(3.4-6.3)	4.4(3.3–6.0)	
Shear wave velocity (m/s)			P < 0.001
Mean ± SD	1.61 ± 0.09	1.43 ± 0.07	
Median(range)	1.61(1.45–1.81)	1.44(1.31–1.55)	
Dispersion slope (m/s/kHz)			P < 0.001
Mean ± SD	13.5 ± 1.39	12.4 ± 1.40	
Median(range)	13.5(11.1–16.7)	12.2(8.9–15.8)	
IQR/median values of m/sec measurements			P = 0.79
Mean ± SD	0.04 ± 0.01	0.04 ± 0.01	
Median(range)	0.04(0.03–0.07)	0.04(0.03–0.06)	
IQR/median values of kPa measurements			P = 0.47
Mean ± SD	0.08 ± 0.02	0.08 ± 0.02	
Median(range)	0.08(0.05–0.13)	0.07(0.05–0.12)	

**Fig. 4:**
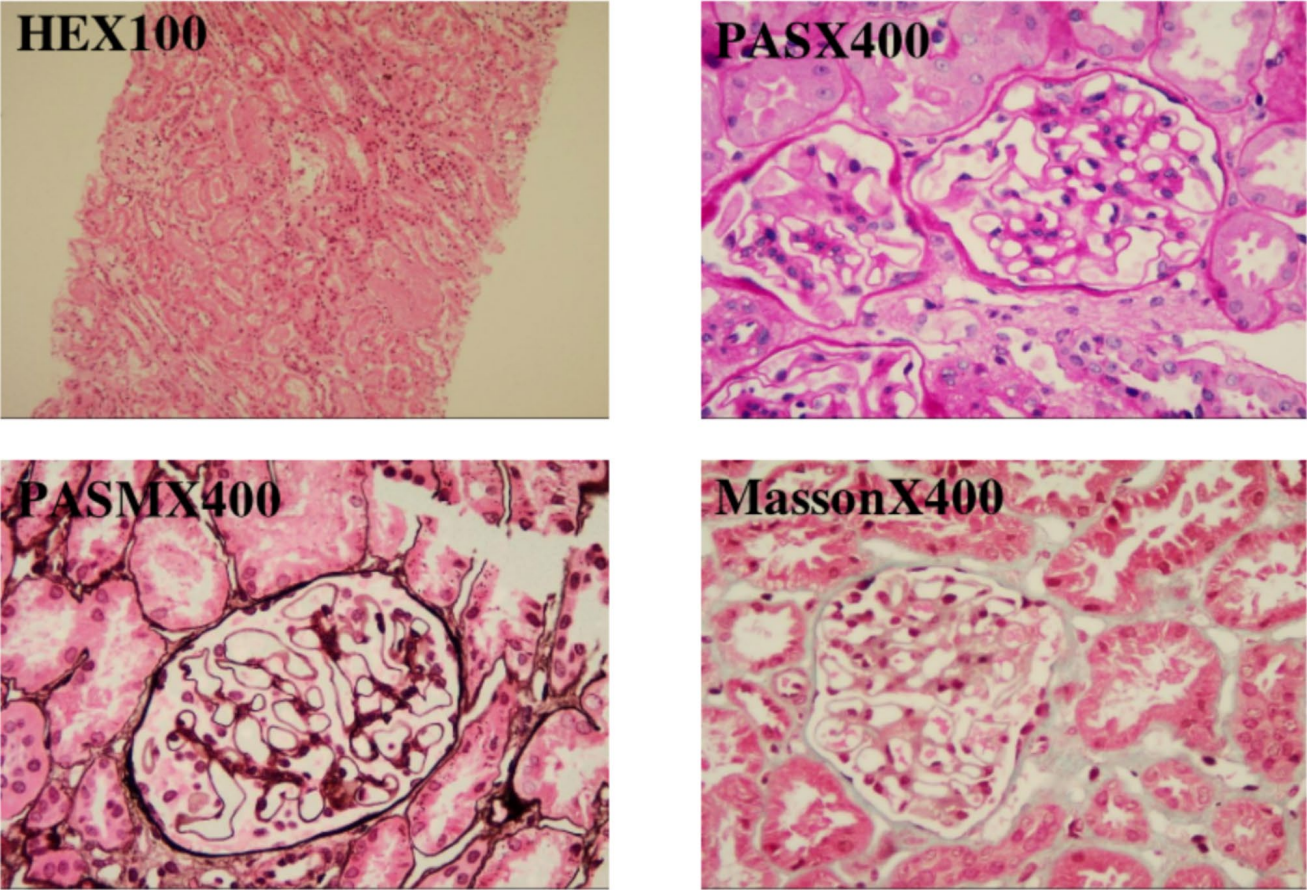
Renal pathology results of a 4-year-old pediatric with Henoch-Schönlein purpura: by HE staining and special staining, it was found that the mesangial cells and stromal stromal were light-to-moderate focal hyperplasia, and the renal interstitium was scattered in inflammatory cell infiltration, without obvious fibrosis

Among the enrolled patients, the pathological findings on percutaneous renal biopsy were minimal change disease (32.6%), focal segmental glomerulosclerosis (11.6%), purpura nephritis (20.9%), lupus nephritis (14.0%), and IgA nephropathy (20.9%) (Table 3; Fig. 4).

**Table 3:** Disease type distribution for the disease group

Disease type distribution		Number of cases
Minimal change disease		14
Focal segmental glomerulosclerosis		5
Purpura nephritis		9
Lupus nephritis		6
IgA nephropathy		9

**Fig. 5:**
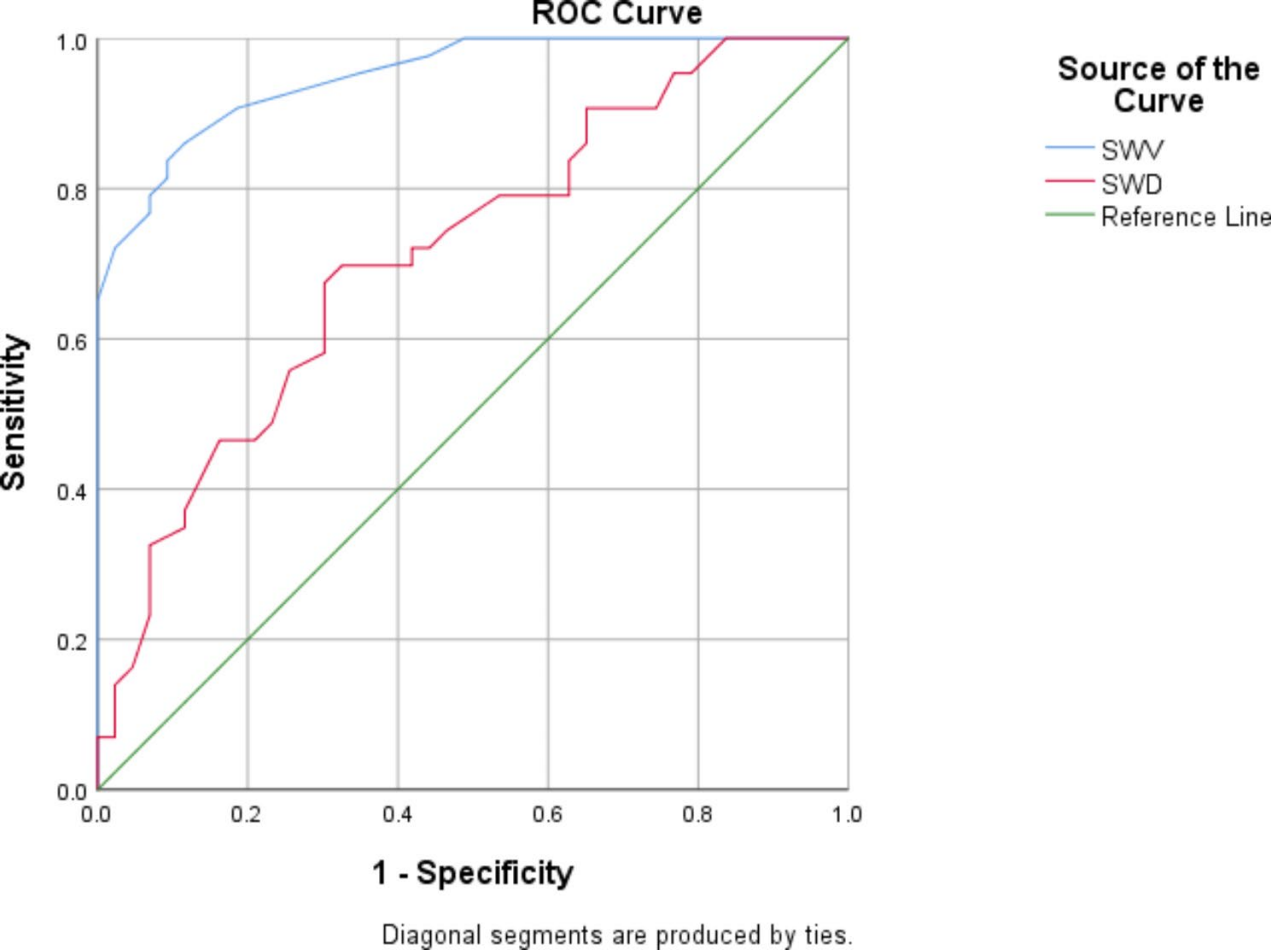
ROC curve of kidney, AUC = 0.95, the SWV cut-off = 1.51 m/s, and the corresponding sensitivity was 83.7% and specificity was 90.7%; AUC = 0.71, the SWD cut-off = 13.1(m/s)/kHz, corresponding to a sensitivity of 67.4% and a specificity of 69.8%

ROC curves were used to analyze the SWV and dispersion slope measurements between the patient and control groups. The results showed that when the area under the curve (AUC) was 0.95, the SWV cut-off value was 1.51 m/s, and the corresponding sensitivity and specificity were 83.7% and 90.7%, respectively (Fig. 5). When the AUC value was 0.71, the SWD value was 13.1 (m/s)/kHz, corresponding to a sensitivity of 67.4% and a specificity of 69.8% (Fig. 5).

Correlation analysis showed that there was no correlation between SWV and occult blood, serum creatinine, 24-h urine protein, blood albumin, age, BMI, sex, and ROI box depth of the patients (p = 0.67, p = 0.61, p = 0.48, p = 0.96, p = 0.33, p = 0.46, p = 0.81 and p = 0.32, respectively). Correlation analysis showed that dispersion slope was not correlated with occult blood, serum creatinine, 24-h urine protein, blood albumin, age, BMI, sex, and ROI box depth of children with glomerular disease (p = 0.74, p = 0.87, p = 0.55, p = 0.26, p = 0.59, p = 0.12, p = 0.62, and p = 0.15, respectively).

**Fig. 6:**
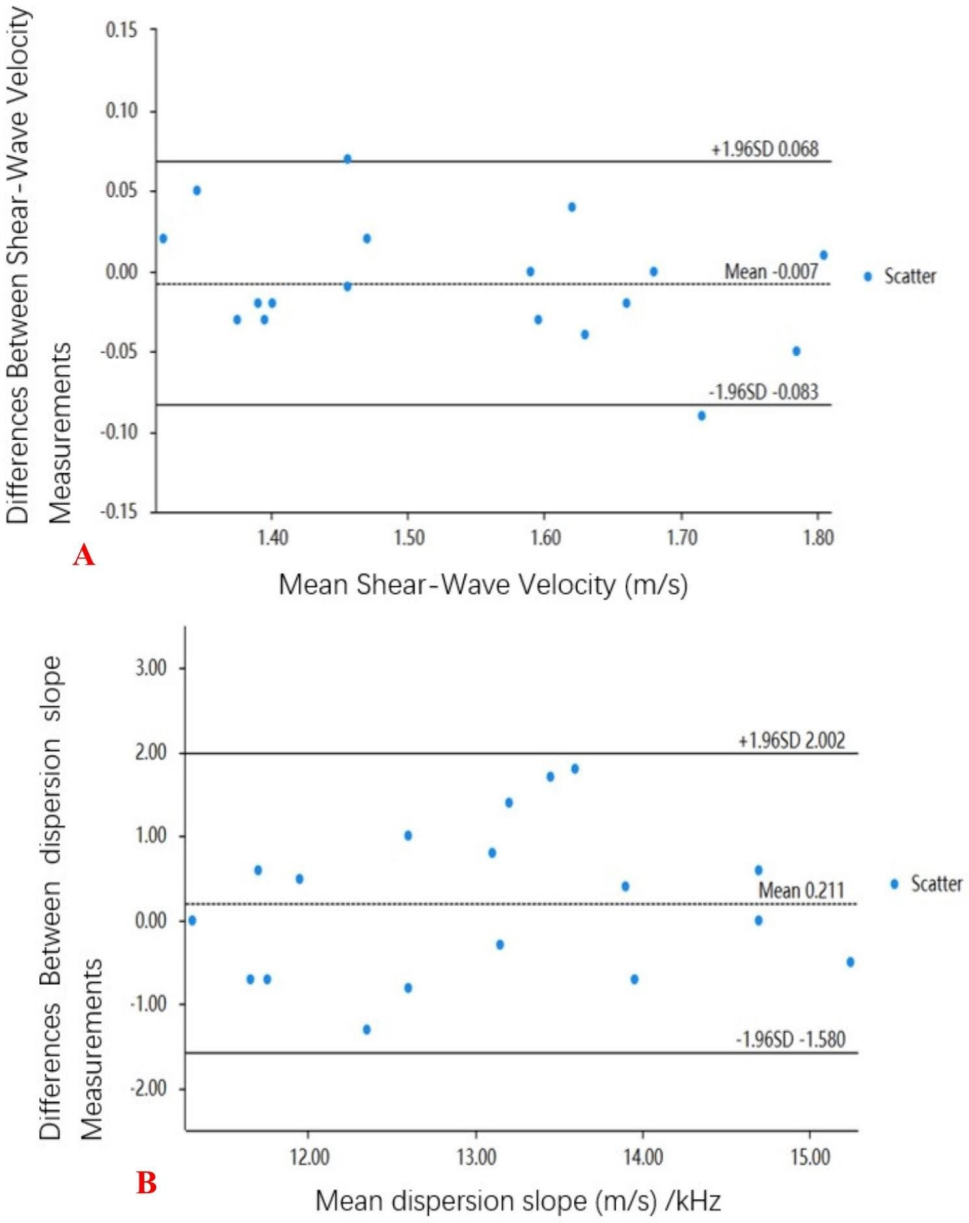
**A**, Bland–Altman plot of variability for Shear-Wave Velocity measurements; **B**, Bland–Altman plot of variability for dispersion slope measurements. Horizontal bolded lines indicate 95% upper and lower limits of agreement and the mean in the middle

The intra-observer agreement of SWV measurements was 0.97(95%CI: 0.93–0.99; P < 0.001), indicating that the SWV measurement had good repeatability. Interobserver variability is shown in Fig. 6 as the Bland-Altman plot. The mean difference for the SWV of the renal between the two subjects was − 0.007 ± 0.038.

The intra-observer agreement of dispersion slope measurements was 0.77 (95%CI: 0.49–0.91; p < 0.001), indicating that the dispersion slope measurements of the kidney had substantial agreement reproducibility. Interobserver variability is shown in Fig. 6 as the Bland-Altman plot. The mean difference for the dispersion slope of the renal between the two subjects was − 0.211 ± 0.914.

## Discussion

To our knowledge, this is the first study to evaluate the viscoelasticity of renal parenchyma in children with glomerular disease using SWE combined with dispersion imaging. Shear wave elastography combined with dispersion imaging showed higher SWV and dispersion slope in the patient group than in the control group when evaluating the viscoelasticity of renal parenchyma. ROC analysis showed that SWV and dispersion slope could distinguish between the patient and control groups, and SWV had higher efficiency. When the AUC was 0.95, the sensitivity, specificity, and cut-off values were 83.7%, 90.7%, and 15.1 m/s, respectively.

Glomerular disease is a common type of diffuse kidney disease in children. During the development of glomerular disease, pathological changes such as glomerulosclerosis, vascular collapse, and atrophy, interstitial fibrosis, and inflammation occur [13], which may lead to changes in the tissue viscoelasticity. Because of conventional ultrasonography is not very sensitive for the detection of early kidney damage, and positive results can only be obtained when the kidneys are significantly damaged, so that the kidneys have progressed to significant parenchymal damage or even insufficiency pathologically [14]. In recent years, the development of new ultrasound technology has provided new ideas for the diagnosis of chronic kidney disease, e.g., SWE has been used to assess SWV in renal interstitial fibrosis in chronic kidney disease [15–17]. The results of this study showed that bilateral renal SWV measurements in the patient group were significantly higher than those in the control group. [16] Previous study [18] showed that the SWV was higher in the glomerular disease group than in the healthy control group, which was consistent with our findings, but only the elasticity of the kidney was examined and its viscosity was not evaluated.

To the best of our knowledge, SWD technology is mostly used in the field of liver [19, 20], and relevant studies had pointed out that there were differences in SWV and dispersion coefficient measured by different inflammatory stages and concluded that SWV was mainly related to liver fibrosis grade, and dispersion slope was mainly related to inflammation and necrosis grade [10]. But this is the first time the technique has been applied in kidney research. In our study, we found that the dispersion slope of children with glomerular disease was higher than those of healthy children (p < 0.001). It is well known that dispersion is related to the slow wave velocity and frequency of slow wave attenuation in the viscous component [11], and the analysis of the dispersion characteristics of shear waves can be used as an indirect method to measure viscosity. Therefore, we believe that the promising results of SWD in glomerular disease may be of value in diagnosis as well as later quantitative monitoring, considering the application of SWD in the assessment of liver disease and its non-invasive nature and ease of access.

ROC curves were used to determine the best SWV and dispersion slope for the diagnosis of glomerular diseases in children. The critical value of SWE for predicting glomerular disease in children was 1.51 m/s, with a sensitivity of 83.7% and specificity of 90.7%, which was close to the critical value of Xu et al.‘s study, but our sensitivity and specificity were better than theirs [18]. This also indicates that SWE has a good diagnostic ability in glomerular diseases in children. The sensitivity and specificity of the cut-off value of SWD suggested by the ROC curve are not strong, which may also be related to the differences in the mechanism, time, and severity of the inflammatory process of kidney diseases. However, it is still necessary to further expand the sample size to conduct controlled studies according to disease severity.

The pathological results of children in the disease group showed that very few children had fibrosis in kidney tissue, which may be related to the age and severity of the disease. This finding shows that changes in kidney stiffness are related to other factors in addition to renal fibrosis. And the study by Gennisson et al. [21] showed that the elasticity of the kidney is related to urine pressure and increases with increased urine pressure. In addition, in studies of glomerular diseases in adults, it has been found that age has an effect on kidney stiffness, since degeneration is a physiological phenomenon of cell and organ aging and is therefore associated with structural changes in the kidneys [22]. Due to these changes, renal blood flow decreases with age, which has an impact on SWV values [23, 24].

To control the quality of the measured SWV, Maralescu et al. [25] found that renal stiffness was influenced by the measurement depth (r=-0.3776, p = 0.0075). Our results showed that the measurement depth of the patient group and the control group was not statistically significant (p = 0.80), which also indicated that the measurement depth did not affect the measurement of SWV and dispersion slope. Further, Barr et al. proposed that interquartile range (IQR) should be used to assess the quality of the data. IQR is a measure of statistical dispersion equal to the difference between the upper and lower quartiles. The IQR/median was < 0.30 for kPa measurements and < 0.15 for m/s measurements, indicating that the dataset is likely to be acceptable [26]. As there are no guidelines in the current literature to ensure appropriate SWD measurements, and dispersal slope measurements also require appropriate SW velocity measurements, the recommended 2D-SWE method should be used [26]. This quality standard was also followed in the SWV measurements in our study.

The current study has some limitations. First, we studied relatively small sample sizes and did not group the included samples by age. Therefore, we need to further expand the sample size and group children of different ages to better compare the SWV and dispersal slope range of the normal renal cortex in children of different ages with the results measured in children of corresponding age with disease. Second, the number of diseases included in the study is small, which will be one of the key points of our next research. In addition, no pathological grading was performed on the included disease groups during the study period; hence, there was no conclusion on whether there were significant statistical differences in SWV and dispersion slope measurements between different pathological grades.

This study suggests that SWE and SWD imaging are likely useful in distinguishing healthy from glomerular disease renal parenchyma based on differences in tissue viscoelastic behavior. Although our findings are preliminary, it suggests that the method has considerable clinical potential.

## Data Availability

The datasets generated and/or analyzed during the current study are not publicly available due to patient privacy protection, but are available from the corresponding author on reasonable request.

## List of Abbreviations

SWE: Shear wave elasticity
SWD: Shear wave dispersion imaging
SWV: Shear waves velocity
CKD: Chronic kidney disease
ROI: Region of interest
AUC: Area under the curve
NAFLD: Nonalcoholic fatty liver disease
BMI: Body mass index
